# Prevalence of Helicobacter pylori infection among gastritis and dyspeptic patients in Jos, Plateau State, Nigeria

**DOI:** 10.3205/dgkh000635

**Published:** 2026-03-02

**Authors:** David Veronica Ekpiwre, Hashimu Zakari, Anayochukwu Chibuike Ngene, Hosea J. Zumbes, Amos Obaje Ogaji, Oluwatoyin Debby Coulthard, John Danjuma Mawak

**Affiliations:** 1 Department of Microbiology, Faculty of Natural Sciences, University of Jos, Jos, Plateau State, Nigeria; 2 Department of Microbiology, College of Natural Sciences, Michael Okpara University of Agriculture, Umudike, Nigeria

**Keywords:** Helicobacter pylori, gastritis, dyspepsia, prevalence, antibiotic resistance, Nigeria

## Abstract

**Background:**

*Helicobacter (H.) pylori* infection remains a major cause of gastritis and dyspepsia globally, with disproportionately high prevalence in developing regions, including parts of Africa where infection rates often exceed 70%. In many of these settings, limited region-specific data on prevalence and antimicrobial resistance hinder effective diagnosis, treatment, and control strategies.

**Objective:**

This study investigated the prevalence, molecular characteristics, and antibiotic susceptibility patterns of *H. pylori* among patients presenting with gastritis and dyspepsia in Jos, Plateau State, Nigeria.

**Methods:**

A total of 136 symptomatic patients were recruited: 36 dyspeptic individuals undergoing endoscopy and 100 gastritis patients providing stool samples. Specimens (gastric biopsies and stool) were initially screened using rapid urease test (RUT) and stool antigen test (SAT). Positive samples underwent culture for isolation, followed by phenotypic identification and polymerase chain reaction (PCR) confirmation with genus- and species-specific primers. Antibiotic susceptibility was evaluated via disk diffusion method.

**Results:**

*H. pylori* prevalence was 66.7% in dyspeptic patients and 48% in gastritis patients. Infection rates were significantly associated with age in both groups (p<0.05), but not with gender. Culture produced 10 presumptive helicobacter isolates; PCR confirmed 6 as helicobacter spp. and 3 as *H. pylori*. Susceptibility testing showed high resistance to ciprofloxacin, clarithromycin, azithromycin, and amoxicillin. All isolates were susceptible to streptomycin, with variable responses to ofloxacin, augmentin (amoxicillin-clavulanate), and septrin (trimethoprim-sulfamethoxazole), suggesting the emergence of multidrug-resistant strains.

**Conclusion:**

The findings reveal a substantial *H. pylori* burden among symptomatic patients in Jos, coupled with alarming resistance to key eradication antibiotics. These results highlight the urgent need for routine molecular diagnostics and ongoing local surveillance of antimicrobial resistance to inform tailored treatment regimens and enhance clinical outcomes.

## Zusammenfassung

**Hintergrund:**

Eine Infektion mit *Helicobacter (H.) pylori* ist weltweit nach wie vor eine der Hauptursachen für Gastritis und Dyspepsie, wobei die Prävalenz in Entwicklungsregionen unverhältnismäßig hoch ist, darunter auch in Teilen Afrikas mit Infektionsraten oft über 70%. In vielen dieser Regionen behindern begrenzte regionsspezifische Daten zur Prävalenz und Antibiotikaresistenz die wirksame Diagnose, Behandlung und Bekämpfungsstrategien.

**Zielsetzung:**

Untersucht werden sollten die Prävalenz, die molekularen Eigenschaften und die Antibiotikaempfindlichkeit von *H. pylori* bei Patienten mit Gastritis und Dyspepsie in Jos im Bundesstaat Plateau, Nigeria.

**Methoden:**

Es wurden 136 symptomatische Patienten rekrutiert, 36 mit Dyspepsie, die sich einer Endoskopie unterzogen, und 100 mit Gastritis, die Stuhlproben zur Verfügung stellten. Die Proben (Magenbiopsien und Stuhl) wurden zunächst mit einem Urease-Schnelltest (RUT) und einem Stuhl-Antigentest (SAT) untersucht. Positive Proben wurden zur Isolierung kultiviert, anschließend phänotypisch identifiziert und mittels Polymerasekettenreaktion (PCR) mit genus- und artspezifischen Primern bestätigt. Die Antibiotikaempfindlichkeit wurde mittels Disk-Diffusionsmethode bewertet.

**Ergebnisse:**

Die Prävalenz von *H. pylori* betrug 66,7% bei Patienten mit Dyspepsie und 48% bei Patienten mit Gastritis. Die Infektionsraten standen in beiden Gruppen in signifikantem Zusammenhang mit dem Alter (p<0,05), jedoch nicht mit dem Geschlecht. Die Kultur ergab 10 mutmaßliche Helicobacter-Isolate; mittels PCR wurden 6 als Helicobacter-Spezies und 3 als *H. pylori* bestätigt. Die Empfindlichkeitstests zeigten eine hohe Resistenz gegenüber Ciprofloxacin, Clarithromycin, Azithromycin und Amoxicillin. Alle Isolate waren empfindlich gegenüber Streptomycin mit unterschiedlichen Reaktionen auf Ofloxacin, Augmentin (Amoxicillin-Clavulanat) und Septrin (Trimethoprim-Sulfamethoxazol), was auf das Auftreten multiresistenter Stämme hinweist.

**Schlussfolgerung:**

Die Ergebnisse zeigen eine erhebliche *H. pylori*-Belastung bei symptomatischen Patienten in Jos, verbunden mit einer alarmierenden Resistenz gegen wichtige Antibiotika zur Eradikation und unterstreichen die dringende Notwendigkeit einer routinemäßigen molekularen Diagnostik und kontinuierlichen lokalen Überwachung der Antibiotikaresistenz, um maßgeschneiderte Behandlungsschemata zu entwickeln und die klinischen Ergebnisse zu verbessern.

## Introduction

*Helicobacter (H.) pylori* infection continues to pose a significant public health threat owing to its close links with chronic gastritis, dyspepsia, peptic ulcers, and gastric cancers [1], [2]. The pathogen spreads mainly via fecal-oral and oral-oral transmission through contaminated food or water, or direct person-to-person contact, which promotes rapid dissemination in areas with poor hygiene and sanitation [3], [4], [5]. Its successful colonization of the gastric environment relies on key adaptive features, such as flagellar motility, urease enzyme activity to counteract stomach acid, and adhesins that enable firm attachment to epithelial cells, thereby sustaining long-term infection and ongoing mucosal inflammation [6], [7].

Worldwide, *H. pylori* affects roughly half the global population, with markedly higher rates (often reaching 80–90%) in low- and middle-income countries, including Nigeria [8], [9]. The bacterium has been designated a class I carcinogen, playing a major role in cancers attributable to infectious agents [10], [11]. Considerable genetic and phenotypic diversity among strains adds complexity to disease manifestation, host responses, and therapeutic success [12].

A pressing issue in treating *H. pylori* is the escalating antimicrobial resistance [13]. Resistance to standard agents in eradication therapies (such as clarithromycin, metronidazole, amoxicillin, and fluoroquinolones) is rising globally, resulting in reduced cure rates and higher recurrence [14]. In resource-limited settings like Nigeria, treatment is often prescribed empirically without localized data on infection rates or resistance profiles, potentially driving the emergence of multidrug-resistant strains and contributing to treatment failures [15], [16].

To address these gaps, localized studies on prevalence, molecular traits, and antibiotic susceptibility of *H. pylori* in symptomatic individuals are essential [17], [18]. This study investigates the infection prevalence among patients with gastritis and dyspepsia, while examining the phenotypic and genotypic properties of isolated strains, with a focus on resistance patterns and multidrug resistance. The results aim to guide evidence-based treatment choices, strengthen antimicrobial stewardship efforts, and optimize clinical management of *H. pylori* infection in the target population.

## Materials and methods

### Study population

A total of 36 dyspeptic patients experiencing upper abdominal pain or discomfort, bloating or fullness after meals, nausea and vomiting, heartburn or acid reflux attending the endoscopy unit of Jos University Teaching Hospital (JUTH) and 100 gastritis patients experiencing severe stomach pain and discomfort, and some with bleeding from the stomach attending Plateau State Specialist Hospital in Jos North LGA of Plateau State of different ages were included.

### Inclusion and exclusion criteria

Patients with either Dyspepsia or gastritis who consented were included. While those with without dyspepsia or gastritis and those with who did not consent were excluded.

### Ethical consideration

Ethical clearance was obtained from the Ethics Committee of the Jos University Teaching Hospital, Jos (REF: JUTH/DCS/IREC/127/XXXI/424). Informed consent was sought and obtained from all participants.

### Isolation of H. pylori from biopsy specimens

Gastric biopsy samples were obtained from 36 dyspeptic patients undergoing endoscopy at the Endoscopy Unit of Jos University Teaching Hospital (JUTH). The specimens were immediately transferred into rapid urease broth for initial screening [19], [20]. Only biopsies yielding a positive rapid urease test were selected for bacterial isolation. These were cultured on brain heart infusion (BHI) agar plates supplemented with 7% sheep blood. The inoculated plates were incubated at 37°C in a microaerobic atmosphere to provide optimal growth conditions. Plates were examined daily for colonial growth starting from day 3 up to day 12.

### Isolation of H. pylori from stool

Fresh fecal samples were collected in a sterile wide-mouthed air-tight containers from 100 gastritis patients. The samples were then inoculated into BHI broth and transported to the laboratory under cold chain (ice packs). The stool samples were first processed using *H. pylori* stool antigen (HpSA) kit. The *H. pylori* positive stool samples were emulsified in BHI broth and then incubated at 37°C for 24 hrs under anaerobic environment specifically in carbon dioxide extinction candle jar. Positive broth samples were then cultured on BHI agar supplemented with antibiotics which included 10 mg vancomycin and 5 mg Amphotericin B with 7% sheep blood. The plates were then incubated at 37°C under anaerobic environment. The agar plates were then checked for growth from day 3 through day 12 [21].

### Preliminary identification of H. pylori

The inoculated plates were incubated in 100% humidity at 37°C for up 3–12 days in microaerophilic condition. Spot test using oxidase was then conducted on the colonies to check for the presence of suspected *H. pylori* before being sub-cultured on nutrient agar. An isolate was identified as *H. pylori* on the basis of positive catalase, oxidase and urease reaction, typical colonial morphology of small, round and greyish colonies and the presence of characteristic curved Gram-negative short rods on Gram Stain [22]. Polymerase chain reaction (PCR) was done to further confirmed the isolates.

### Gram staining

On a clean grease free slide, a small drop of normal saline was placed. Using a sterile wire loop a discreet colony from the cultured plate was picked and emulsified on the slide to make a thin smear and was allowed to air-dry. The smear was heat fixed using dry heat. The heat fixed smear was stained with crystal violet (primary stain) and allowed to stay for 60 seconds. The smear was rinsed with water and floated with Lugol's iodine solution (mordant) then allowed for 30 seconds. The iodine was rinsed off with water. Using acetone (decolorizing agent), the smear was flooded for 5 seconds and rinsed immediately under running water. This generates the differential aspect of Gram staining. Finally, smear was counter-stained with safranin (secondary stain) for 60 seconds, rinsed off under running water and the slide was allowed to air-dry. The slide was viewed under the microscope using ×100 oil immersion objectives. *H. pylori* were seen as Gram-negative curve rods [23].

### Oxidase test

A drop of 1% oxidase reagent was applied to a strip of filter paper. A portion of a colony from the culture plate was collected using a sterile wire loop and rubbed onto the reagent-impregnated area. Development of a deep purple coloration within 20 seconds signified a positive result [23].

### Catalase test

On a clean, grease-free glass slide, a colony from the culture plate was transferred using a sterile inoculating loop and smeared across the surface. A drop of 3% hydrogen peroxide was added to the smear and gently mixed. The immediate formation of gas bubbles indicated a positive catalase reaction [23].

### Urease test

Using a flamed wire loop, a colony was picked from the culture plate and stabbed on urease agar slant inside test tube, it was covered and incubated at 37°C for 72 hrs. A color change was observed from orange to pink following incubation which indicated positive test [23]. 

### Maintenance of H. pylori

After purification and identification of the clinical isolates of *H. pylori*, strains were preserved in a small screw capped tube in BHI broth at –4°C.

### Antibiotic susceptibility testing

Antibiotic susceptibility testing was conducted using the disk diffusion method, in accordance with previously described protocols [24], [25] and the Clinical and Laboratory Standards Institute (CLSI) guidelines [24]. A single isolated colony was emulsified in peptone water and adjusted to a turbidity matching the 0.5 McFarland standard. The bacterial suspension was then evenly inoculated onto BHI agar plates. Antibiotic disks were applied to the agar surface, and the plates were incubated under microaerobic conditions at 37°C for 72 hrs. Inhibition zone diameters were measured and interpreted to classify isolates as susceptible, intermediate, or resistant. The antibiotics tested, along with their disk potencies, included ofloxacin (tarivid, 10 µg), trimethoprim-sulfamethoxazole (septrin, 30 µg), chloramphenicol (30 µg), nalidixic acid (30 µg), ciprofloxacin (10 µg), amoxicillin (10 µg), amoxicillin-clavulanate (augmentin, 30 µg), gentamicin (10 µg), pefloxacin (preflacine, 10 µg), and streptomycin (30 µg) (agents frequently employed in standard *H. pylori* eradication regimens).

### Molecular identification

Presumptive *H. pylori* isolates were subjected to confirmatory identification via polymerase chain reaction (PCR) analysis. Genomic DNA was extracted from phenotypically identified* H. pylori* isolates using the QIAamp DNA Mini Kit (Qiagen), in accordance with the manufacturer’s instructions. Briefly, *H. pylori* colonies were collected using a sterile plastic loop and suspended in 200 µL of phosphate-buffered saline (PBS) in sterile 1.5 mL microcentrifuge tubes. The suspensions were vortex-mixed and centrifuged at 8,000 rpm for 1 minute. The supernatants were discarded, and the pellets were resuspended in a fresh 200 µL volume of PBS by vortexing. The resuspended cells were transferred into new sterile 1.5 mL microcentrifuge tubes containing 20 µL of proteinase K, followed by the addition of 200 µL of Buffer AL. The mixtures were thoroughly vortexed for 15 seconds and incubated at 56°C for 10 minutes to achieve cell lysis. The tubes were then briefly centrifuged at 8,000 rpm to remove condensation from the lids. The lysates were applied to QIAamp Mini spin columns placed in 2 mL collection tubes and centrifuged at 8,000 rpm for 1 minute. The columns were transferred to fresh collection tubes, washed with 500 µL of Buffer AW1, and centrifuged at 8,000 rpm for 1 minute, after which the flow-through was discarded. A second wash was performed using 500 µL of Buffer AW2, followed by centrifugation at 14,000 rpm for 3 minutes. To prevent buffer carryover, an additional dry spin was performed at 14,000 rpm for 1 minute. Finally, the spin columns were placed into sterile 1.5 mL microcentrifuge tubes, and 200 µL of Buffer AE (elution buffer) was added directly onto the membrane. After incubation at room temperature for 1 minute, the columns were centrifuged at 8,000 rpm for 1 minute to elute the DNA. The extracted DNA was used immediately for PCR amplification.

### PCR amplification and gel electrophoresis

The following primers were used: HELF: 5’-AACGATGAAGCTTCTAGCTTGCTA-3’, HELR: 5’-GTGCTTATTCSTNAGATACCGTCAT-3’ [17], HPYF: 5’-GCGACCTGCTGGAACATTAC-3’ and HPYR: 5’-CGTTAGCTGCATTACTGGAGA-3’ [18]. Amplification of bacterial DNA was done using 25 µl total reaction volume containing 12.5 µl of 2X master mix, 1 µl of forward primer, 1 µl reverse primer and, 5.5 µl of nuclease-free water and 5 µl of DNA each for *Helicobacter* spp. specific (HEL) and *H. pylori* specific (HPY) detections. The thermocycling profile for HEL was as follows: Initial denaturation at 94°C for 5 min, followed by 35 cycles of denaturation at 94°C for 1 min, annealing at 56°C for 1 min and extension at 72°C for 1 min, held for final extension at 72°C for 10 min. The expected band size was 399 bp. For HPY, each reaction mixture was amplified for 35 cycles as follows: 30 sec at 94°C, 30 sec at 55°C and 30 sec at 70°C. A preincubation of 5 min at 94°C and final extension of 72°C for 30 sec were performed. The expected band size was 138 bp. The amplified products were visualized on 1.5% agarose gel stained with 8 µl ethidium bromide and was ran for 35 min at 100 volts.

## Results

The overall prevalence of *H. pylori* in this dyspeptic cohort was 66.7% (24 out of 36 patients) (Table 1 (Tab. 1)). A significant association was found between age group and *H. pylori* occurrence (Χ²=7.679, p-value=0.022). The infection rate was highest in the 30–39 years age group (85%, 17 out of 20), followed by the 40–49 years group (66.7%, 2 out of 3). The lowest prevalence was observed in the 50–59 years age group (38.5%, 5 out of 13). In contrast, no statistically significant association was found between gender and *H. pylori* infection (Χ²=0.056, p-value=0.813). 

Table 2 (Tab. 2) presents the distribution of *H. pylori* prevalence in stool specimens from gastritis patients, stratified by age and gender (n=100). The overall infection rate was 48%. A statistically significant association was observed between age and *H. pylori* positivity (Χ²=14.336, p=0.006). The highest prevalence occurred in the 50–59 years age group (76.2%; 16/21), followed closely by the 20–29 years group (61.9%; 13/21). In contrast, the lowest rate was recorded among patients aged 60 years and older (27.3%; 6/22). Regarding gender, prevalence was higher among females (52.8%; 38/72) than males (35.7%; 10/28), although complete statistical details for this comparison were not specified in the table.

The occurrence of *H. pylori* and other isolates of the culture medium from dyspeptic and gastric patients is presented in Table 3 (Tab. 3). A total of 75 isolates were obtained of six different genera were identified. *Escherichia (E.) coli *had the highest occurrence at 36%, followed by Pseudomonas species, which accounted for 26.7% of the isolates. *H. pylori* appeared in 13.3% of samples, while yeast cells were detected in 10.7% of the samples. Bacillus species showed a lower frequency of 8%, and Acinetobacter species had the least occurrence at 5.3%.

### Molecular confirmation of presumptive Helicobacter species isolates

PCR analysis using *Helicobacter* genus-specific primers (HELF/HELR) produced the expected amplicon of approximately 399 bp in six of the ten presumptive isolates, confirming their identity as *Helicobacter* species. The molecular weight marker showed clear reference bands, while the negative control exhibited no amplification, indicating the absence of contamination or nonspecific PCR products. The remaining four isolates showed no detectable bands and were therefore considered PCR-negative. Overall, the results demonstrate that 60% of the presumptively identified isolates were molecularly confirmed as *Helicobacter* species using genus-specific PCR. 

### Molecular confirmations of presumptive H. pylori isolates

PCR amplification using *H. pylori*-specific primers (HPYF/HPYR) yielded the expected 138 bp product in three of the six *Helicobacter* genus-positive isolates, confirming them as *H. pylori*. The molecular size marker showed appropriate reference bands, while the negative control showed no amplification, indicating assay specificity and the absence of contamination. The remaining three isolates showed no detectable 138 bp band and were therefore not confirmed as *H. pylori*.

### Antibiotic susceptibility

All confirmed *H. pylori* isolates were resistant to ciprofloxacin, azithromycin, and amoxicillin. Complete susceptibility (100%) was observed with streptomycin, which demonstrated strong inhibitory activity against all isolates. Two isolates were susceptible to ofloxacin, while only one isolate showed susceptibility to fluoroquinolones, clarithromycin, and amoxicillin-clavulanate. Gentamicin showed no activity against any of the isolates. Overall, the results indicate the presence of multidrug-resistant *H. pylori* strains, with streptomycin remaining the most consistently effective agent among those tested.

## Discussion

### Prevalence of H. pylori infection in relation to age and gender

This study provides important insights into the epidemiology of *H. p*ylori infection among dyspeptic and gastritic patients, highlighting both shared global patterns and distinct local dynamics. The observed prevalence of *H. pylori* infection was higher among dyspeptic patients (66.7%) than among gastritis patients assessed via stool antigen testing (48%). This difference is clinically meaningful and aligns with the established role of* H. pylori* as a major etiological agent of dyspeptic symptoms, including chronic gastritis and peptic ulcer disease [26]. The substantial prevalence observed in the broader gastritic population, although lower, further confirms that *H. pylori* remain a key contributor to gastric pathology, even though other infectious and non-infectious factors may also play a role. These findings support current clinical recommendations advocating a “test-and-treat” strategy in symptomatic patients to enable targeted eradication therapy and reduce unnecessary invasive procedures [27].

Age was significantly associated with *H. pylori* infection in both patient groups, underscoring the influence of host age on infection dynamics. Among dyspeptic patients, the highest prevalence was recorded in the 30–39-year age group, followed by a progressive decline in older age groups. This pattern likely reflects the natural history of *H. pylori* infection, whereby acquisition commonly occurs in childhood, but clinical manifestations often become apparent in early to mid-adulthood as cumulative mucosal damage and chronic inflammation progress [28]. The reduced prevalence observed in individuals aged 50–59 years may be partially explained by a birth cohort effect, whereby older individuals experienced lower rates of childhood acquisition due to gradual improvements in hygiene, sanitation, and living conditions over time [29], [30].

In contrast, the gastritic patient cohort exhibited the highest prevalence in the 50–59-year age group, a finding of particular concern given the strong association between long-standing *H. pylori* infection and severe gastric sequelae, including gastric atrophy, intestinal metaplasia, and gastric carcinoma [31]. The marked decline in prevalence among individuals older than 60 years further supports the birth cohort hypothesis, suggesting reduced exposure in earlier decades. Notably, the secondary peak observed among younger adults (20–29 years) indicates ongoing transmission within the community, highlighting persistent public health challenges related to sanitation, water quality, and overcrowding [32].

Gender-related analysis revealed differing patterns between the two patient groups. The absence of a statistically significant gender difference among dyspeptic patients suggests that susceptibility to symptomatic *H. pylori* infection may not be inherently sex-dependent. However, the higher prevalence observed among females in the gastritic cohort is noteworthy. While this finding may partly reflect differences in healthcare-seeking behavior, emerging evidence suggests that hormonal factors, particularly estrogen, may influence gastric mucosal immunity and *H. pylori* colonization density [33]. Additionally, sex-specific variations in gut microbiome composition, which can interact with *H. pylori* and modulate inflammatory responses, may contribute to the observed disparity [34]. These observations underscore the need for sex-stratified analyses in future epidemiological and mechanistic studies.

### Occurrence of H. pylori and other microorganisms in dyspeptic and gastritic patients

Culture-based identification yielded *H. pylori* in only 10 of the 75 isolates obtained, suggesting a relatively low recovery rate. This finding contrasts with reports of culture positivity rates ranging from 40% to 80% in gastric biopsy samples [35], [36] and with Nigerian studies reporting prevalence rates exceeding 80% using molecular techniques [37], [38]. The lower isolation rate observed in this study may be attributable to several factors, including prior exposure to antibiotics or proton pump inhibitors, delays in sample transport, stringent growth requirements of *H. pylori*, and inherent limitations of culture-based methods. These challenges reinforce the importance of integrating culture with more sensitive diagnostic tools such as PCR and rapid urease tests to improve detection accuracy [39].

The predominance of *E. coli* among non-*H. pylori* isolates is consistent with its status as dominant component of intestinal microbiota and its recognized public health relevance, particularly with respect to extended-spectrum β-lactamase (ESBL)-producing strains [40]. Similarly, the recovery of Pseudomonas species supports growing evidence that *Pseudomonas aeruginosa* may be more frequently involved in gastrointestinal colonization and disease than previously appreciated [41]. The detection of Bacillus species likely reflects transient environmental contamination, while the presence of yeast cells aligns with recent findings highlighting the gut mycobiome as an integral component of gastrointestinal microbial ecosystems [42].

### Molecular identification of Helicobacter species and H. pylori

Molecular analysis revealed notable discrepancies between phenotypic identification and PCR confirmation, with only 6 of 10 presumptive Helicobacter isolates confirmed at the genus level. This finding underscores the limited specificity of culture and biochemical methods and highlights the superior accuracy of PCR-based diagnostics for Helicobacter detection [43]. Furthermore, only half of the PCR-confirmed Helicobacter isolates were identified as *H. pylori*, reflecting the diversity of the Helicobacter genus and the potential involvement of non-pylori species such as *H. heilmannii* and *H. felis* in gastric colonization and disease [44]. These findings emphasize the need for precise molecular characterization to fully elucidate the epidemiology and pathogenic relevance of Helicobacter species in clinical settings.

### Antibiotic susceptibility patterns

The antibiotic susceptibility profiles reveal concerning resistance trends across multiple antibiotic classes. Resistance to fluoroquinolones, including ciprofloxacin and ofloxacin, was prominent, consistent with global reports linking widespread antibiotic use to increasing fluoroquinolone resistance in *H. pylori* [45]. Similarly, high resistance rates to macrolides such as clarithromycin and azithromycin are particularly troubling, given the central role of these agents in standard eradication regimens. These findings corroborate reports of declining clarithromycin efficacy and underscore the need for resistance-guided therapy [46], [47].

Beta-lactam antibiotics demonstrated variable susceptibility patterns, suggesting heterogeneity in resistance mechanisms among circulating strains. Such variability highlights the importance of local antimicrobial surveillance to inform empirical treatment decisions and optimize eradication outcomes. In contrast, aminoglycosides, particularly streptomycin, showed high efficacy against the tested isolates, consistent with previous studies reporting low resistance rates to this class [48]. Sulfonamides exhibited moderate activity, indicating potential utility in selected cases, although susceptibility testing remains essential to ensure therapeutic success. Overall, the wide variation in zones of inhibition observed across isolates reflects substantial strain-specific differences in resistance profiles. This heterogeneity underscores the growing challenge posed by multidrug-resistant *H. pylori* and reinforces the need for routine susceptibility testing and tailored treatment strategies to improve eradication rates and limit further resistance development.

Public health interventions aimed at improving sanitation, access to clean water, and awareness of *H. pylori* transmission routes are also recommended to reduce infection rates.

### Study limitations

This study has several limitations that should be considered when interpreting the findings. First, the relatively small number of *H. pylori* isolates confirmed by molecular methods and subjected to antibiotic susceptibility testing may limit the generalizability of the resistance patterns observed. Second, reliance on culture-based identification may have underestimated the true prevalence of *H. pylori*, given its fastidious growth requirements and the potential effects of prior antibiotic or proton pump inhibitor use among participants. Third, the cross-sectional design of the study precludes assessment of causal relationships, temporal trends, and treatment outcomes. In addition, virulence determinants and specific resistance-associated gene mutations were not investigated, which limits insights into strain pathogenicity and molecular mechanisms underlying antimicrobial resistance.

Based on the findings, larger multicenter studies incorporating increased sample sizes are recommended to provide more representative prevalence and resistance data. The integration of advanced molecular techniques, including detection of virulence genes and resistance-associated mutations, would enhance understanding of strain diversity and therapeutic failure. Finally, longitudinal studies evaluating treatment outcomes and reinfection rates are needed to inform optimized management strategies and policy development.

## Notes

### Author’s ORCID 

Ngene AC: https://orcid.org/0000-0003-4730-2834

### Ethical approval 

Ethical clearance was obtained from the Ethics Committee of the Jos University Teaching Hospital.

### Funding

This research was supported by the University of Jos through the Institutional-Based Research (IBR) Grant, Grant No. 036.

### Competing interests

The authors declare that they have no competing interests.

## Figures and Tables

**Table 1:**
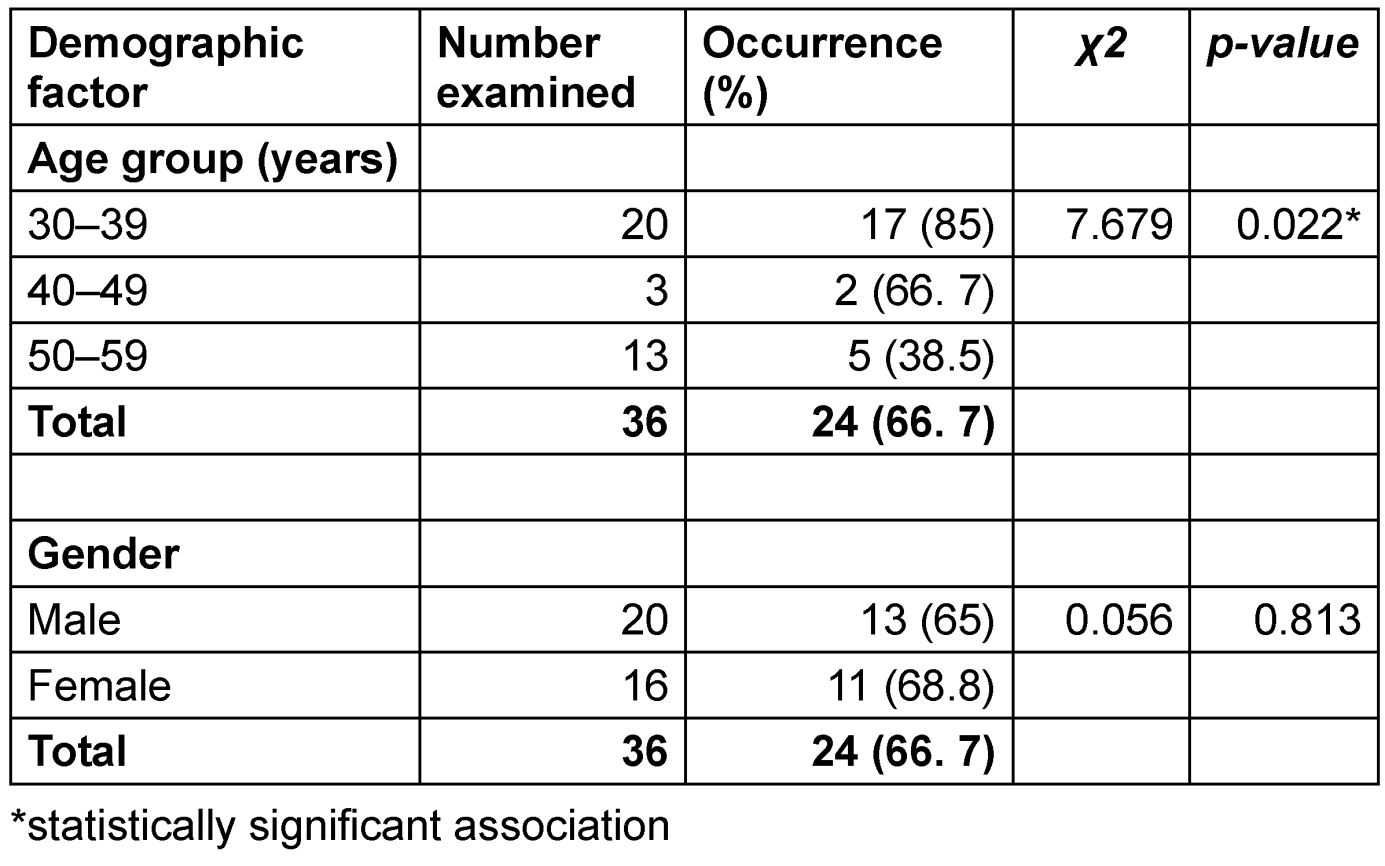
Occurrence of *H. pylori* in cases of dyspeptic patients in relation to age and gender (n=36)

**Table 2:**
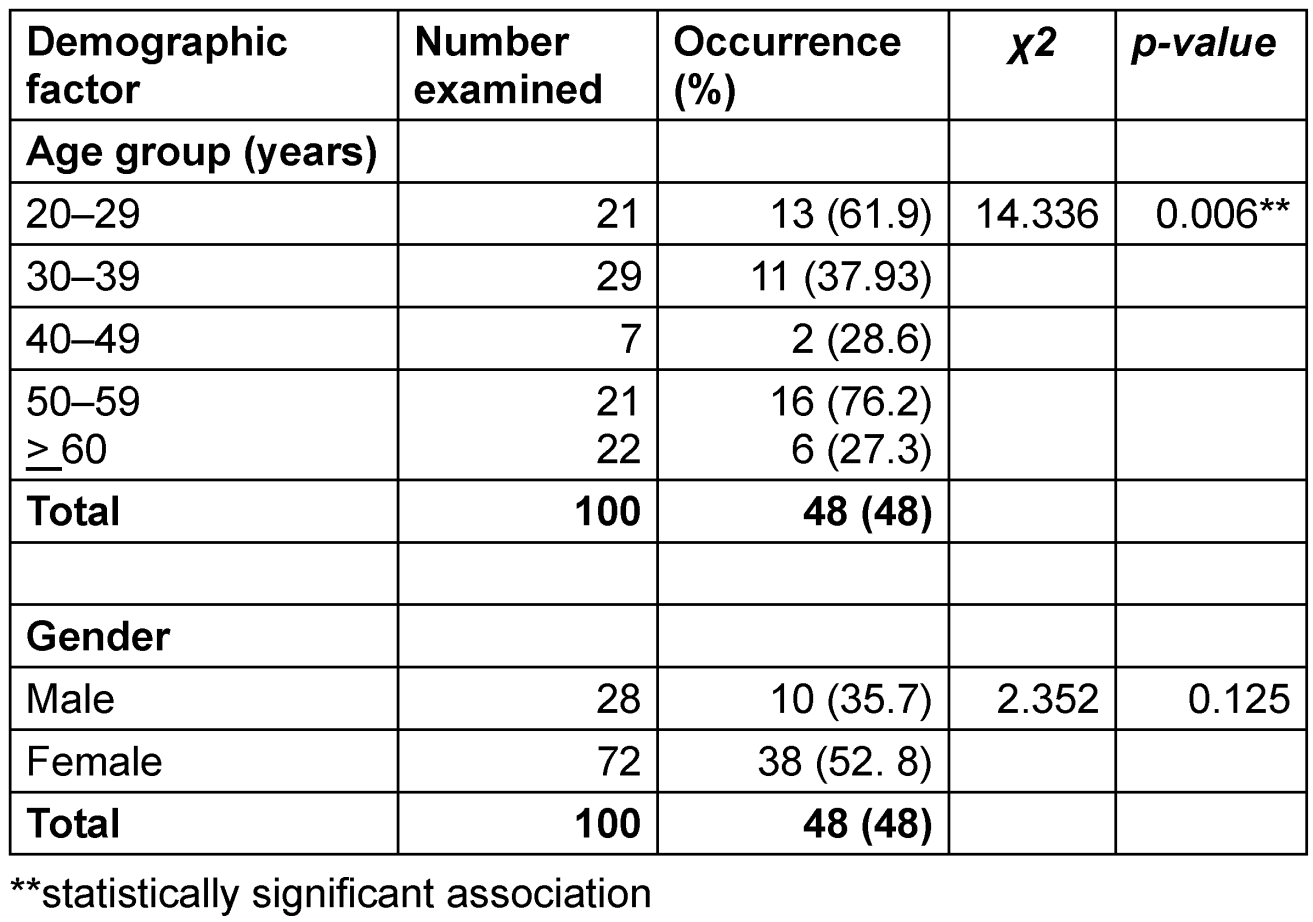
Prevalence of *H. pylori* in the stool of gastric patients in relation to age and sex (n=100)

**Table 3:**
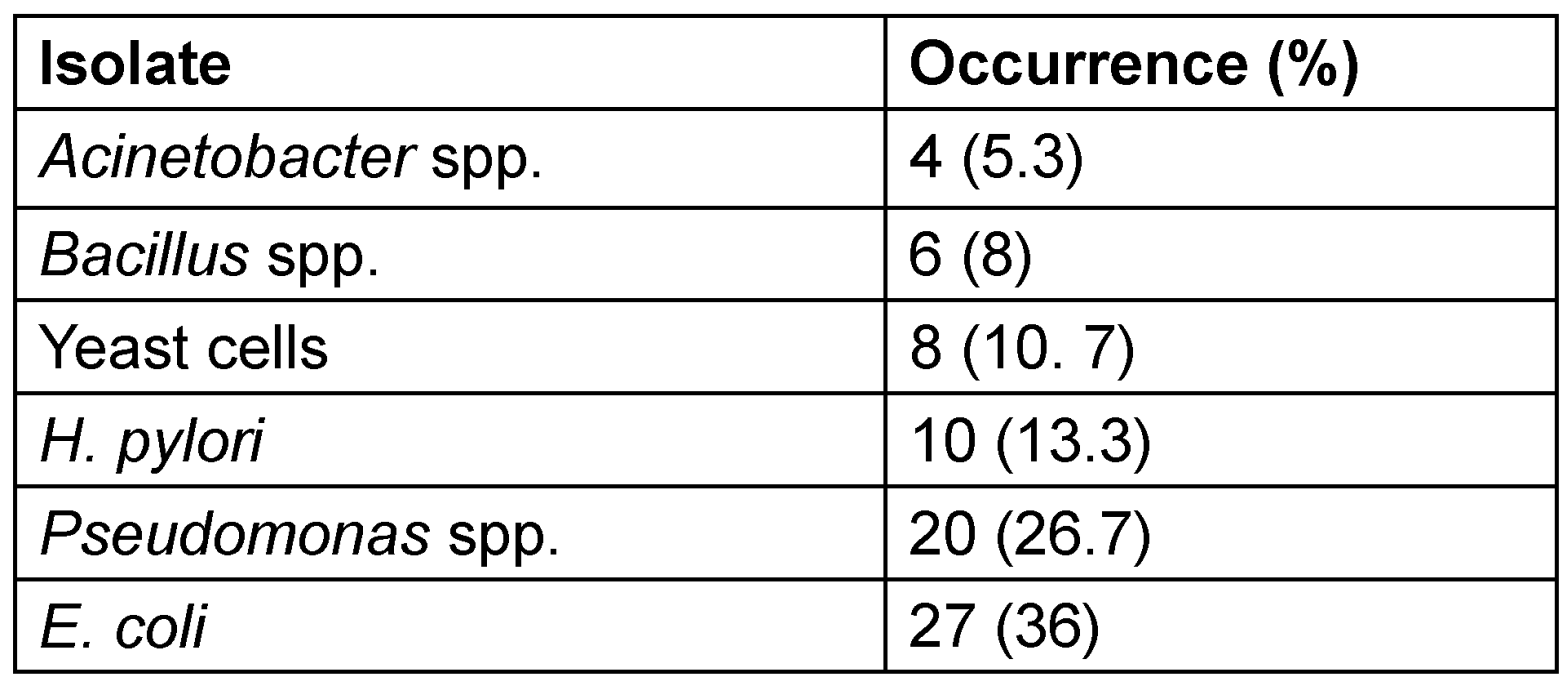
Occurrence of *H. pylori* and other organisms in dyspeptic and gastric patients (n=75)
